# The promising alliance of anti-cancer electrochemotherapy with immunotherapy

**DOI:** 10.1007/s10555-016-9615-3

**Published:** 2016-03-18

**Authors:** Christophe Y. Calvet, Lluis M. Mir

**Affiliations:** Vectorology and Anticancer Therapies, UMR8203, CNRS, Univ. Paris-Sud, Gustave Roussy, Université Paris-Saclay, 94805 Villejuif, France

**Keywords:** Electrochemotherapy, Electrogenetherapy, Immunotherapy, Cancer, Metastasis, Immunity

## Abstract

Anti-tumor electrochemotherapy, which consists in increasing anti-cancer drug uptake by means of electroporation, is now implanted in about 140 cancer treatment centers in Europe. Its use is supported by the English National Institute for Health and Care Excellence for the palliative treatment of skin metastases, and about 13,000 cancer patients were treated by this technology by the end of 2015. Efforts are now focused on turning this local anti-tumor treatment into a systemic one. Electrogenetherapy, that is the electroporation-mediated transfer of therapeutic genes, is currently under clinical evaluation and has brought excitement to enlarge the anti-cancer armamentarium. Among the promising electrogenetherapy strategies, DNA vaccination and cytokine-based immunotherapy aim at stimulating anti-tumor immunity. We review here the interests and state of development of both electrochemotherapy and electrogenetherapy. We then emphasize the potent beneficial outcome of the combination of electrochemotherapy with immunotherapy, such as immune checkpoint inhibitors or strategies based on electrogenetherapy, to simultaneously achieve excellent local debulking anti-tumor responses and systemic anti-metastatic effects.

## Introduction

Many methods have been developed for *in vitro* and *in vivo* applications to deliver molecules inside the cells. Although very efficient, viral methods are rather restricted to nucleic acid transfer and have brought many safety concerns, while chemical methods lack efficiency for localized *in vivo* applications. Among the physical methods, electroporation has raised a great excitement over the last two decades [1, 2]. Indeed, the use of electricity to manipulate cells or target tissues appears quite seducing. Electroporation is increasingly being used among the scientific and the medical communities, as it is a safe and efficient technique to transfer a variety of material (e.g., nucleic acids, cytotoxic drugs, and ions) into target cells and tissues without harming them. This review will first give the readers an overview of electroporation-based therapeutic strategies and will then highlight the rationale of combining them with immunotherapy in the context of anti-cancer treatments.

## Insights into cell electroporation and its medical applications

Pioneer works related to electroporation (also called electropermeabilization) started in 1968 with Sale and Hamilton who showed that the application on cells of intense electric fields induces the release of intracellular molecules that are unable to cross by themselves the cell membrane under physiological conditions [3]. Nearly 15 years later, Neumann and collaborators managed to transfer exogenous molecules (i.e., DNA) into mouse lyoma cells using electroporation [4], thereby giving rise to a wide range of applications for this technology.

**Fig. 1:**
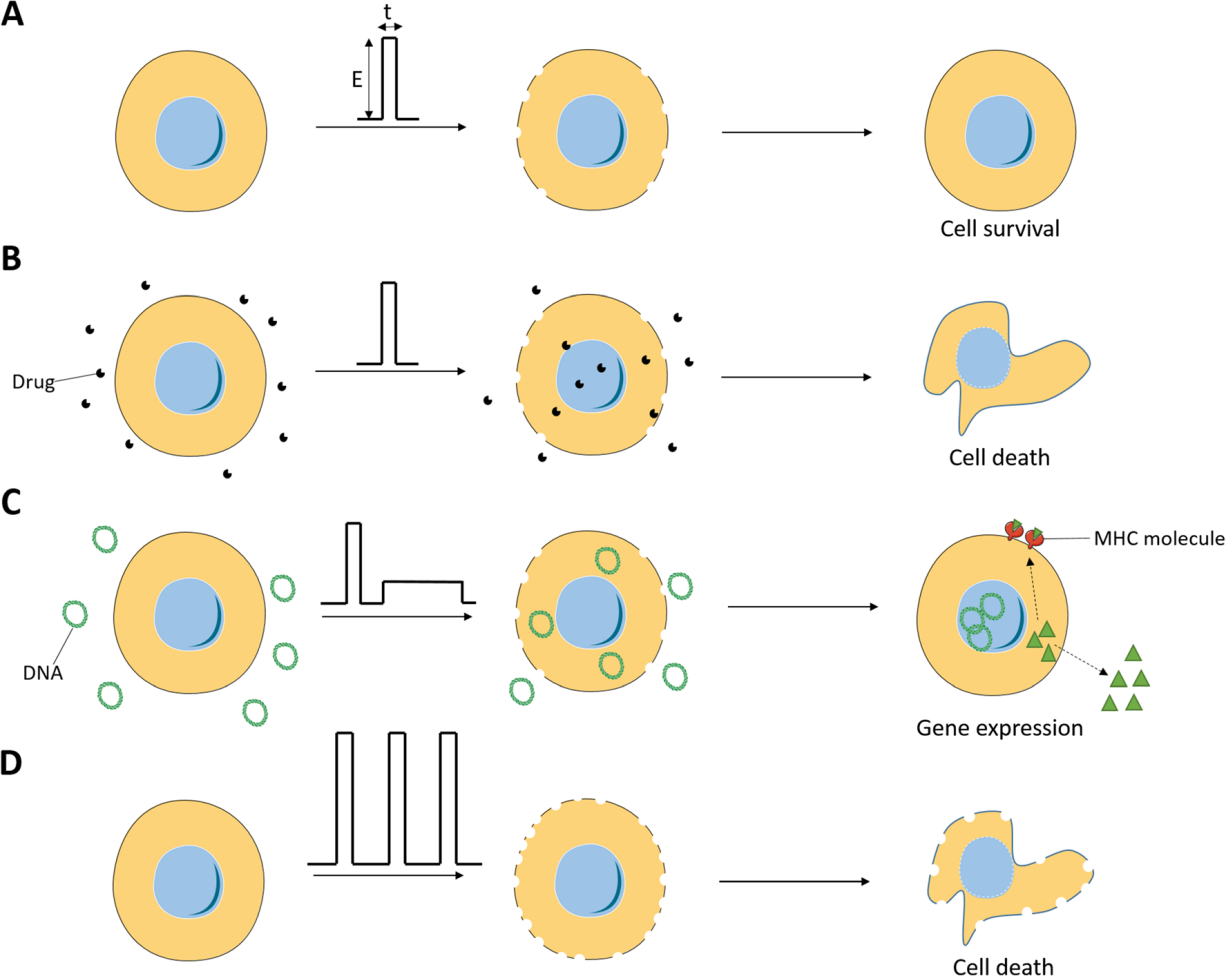
Principle of biomedical applications of electroporation. **a** Electroporation consists in the delivery of a limited number of short and intense electric pulses which are defined by an intensity *E* and a duration *t*. Above a certain threshold of the *E* and/or *t* parameters, cell membrane defects appear and result in cell permeabilization. After a given lag time, cell membrane integrity is restored leading to cell survival. **b** Electrochemotherapy consists in the delivery of short and intense electric pulses following the administration of non- or low-permeant cytotoxic drugs, such as bleomycin. Cell membrane permeabilization permits the drug to enter the target cells and eventually to trigger cell death through multiple DNA breaks, which are lethal for dividing cells. **c** Electrogenetherapy relies on gene electrotransfer, namely, the conjunction of DNA delivery and electroporation. Gene electrotransfer can be achieved by first permeabilizing the cell membrane thanks to short and intense electric pulse deliveries and second by driving electrophoretically the DNA toward the electroporated membrane thanks to a long and low-voltage electric pulses. It is then expected that a protein of interest is produced and epitope presentation occurs on MHC molecules. **d** Irreversible electroporation consists in the use of excessive electroporation to cause cell death. Different approaches can lead to this outcome including the use of very long or very intense electric pulses or the use of too many of electric pulses whose characteristics are similar to those used in viability-preserving electroporation strategies

Electroporation is a generic term used to describe the phenomenon of increased permeability of the cell membrane following the application of short and intense electric pulses (EPs). Here, in Sects. 2–5, we will discuss the biomedical applications of electric fields preserving cell viability (Fig. 1a). The achievement of membrane electroporation depends on the biophysical properties of the cell membrane and those of the cell-surrounding medium [1]. Although intense researches are being conducted to understand the mechanisms underlying membrane electroporation, these are still speculative.

Even though electroporation is a promising approach for the treatment and the prevention of several pathologies, cancer is nowadays the major indication of electroporation-based therapies [2]. The following three different medical applications of electroporation have already been developed and brought to clinics:

- Electroporation to transfer drugs and small molecules (Fig. 1b), the anti-cancer electrochemotherapy (ECT; see Sect. 2) is the combination of electroporation and cytotoxic drugs that do not freely cross the plasma membrane. ECT was the first application that reached the clinical stage [5, 6]. ECT works very efficiently and without major side effects. It selectively kills the tumor cells and spares the normal non-dividing cells in the volume exposed to the electroporating EPs (usually trains of eight short pulses of 100-μs duration). However, ECT remains a local treatment with no obvious effects on distant metastases.
- Electroporation to transfer nucleic acids inside the cells (Fig. 1c), the electroporation-based gene transfer, that, if a therapeutic outcome is desired, constitutes a subcategory of gene therapy; the electroporation-mediated gene therapy, namely, electrogenetherapy (EGT; see Sect. 3), is an approach rapidly expanding in cancer and non-cancer therapeutic domains [7, 8]. It covers some of the classical gene therapy objectives with no virus use. The EPs used are often longer (tens of milliseconds), and combinations of short and long EPs are also delivered. In the anti-cancer field, the two most exciting EGT applications are cytokine therapy and non-viral DNA vaccination, both at the crossing of electroporation and immunology.
- Electroporation to kill cells (Fig. 1d), the irreversible electroporation (IRE) is also developing for liquid sterilization in the food and environment industries, among other uses [2]. In this application, what is sought is cell death triggering due to excessive electroporation. This local ablative treatment is not selective against the tumor cells, killing also the normal cells in the volume of tissue exposed to the EPs. Because of its absence of selectivity, IRE is not developed in this review.

The use of very short EPs of a duration of tens to hundreds of nanoseconds is also being explored [2], with purposes (e.g., manipulation of internal cell membranes and cell death induction) which are also beyond the scope of this article.

Section 4 of this review highlights the preclinical data showing the potential synergistic effect of the combination of ECT with immunotherapy strategies, including those based on EGT. In Sect. 5, we report recent clinical data suggesting that the promising alliance of anti-cancer electrochemotherapy with immunotherapy is already coming to the reality.

## Electrochemotherapy

As a means to increase the uptake of cytotoxic drugs by cancer cells, electroporation is used in the context of anti-tumor ECT.

### Bases of ECT

ECT is a non-thermal and non-ablative local treatment of solid tumors consisting in the application of EPs combined with the administration of non-/low-permeant anti-cancer molecules [2, 9, 10]. The proof of concept of ECT has been made in many *in vitro* and *in vivo* models, and the treatment is now routinely applied in humans.

The EPs, locally delivered to the whole volume of the nodule, are meant to reversibly permeabilize the cells located in the treated region, without killing them. The anti-cancer drug, administered directly into the tumor or systemically, can then enter the electroporated target cells without restrictions to achieve its cytotoxic activity (Fig. 1b).

The main advantage of ECT over other chemical-based anti-cancer treatments is to selectively target tumor cells, on the one hand, by applying EPs locally, and on the other hand, by using an anti-cancer drug displaying specific cytotoxicity toward dividing cells, which are mainly the cancer cells. Two conditions are required for ECT to be efficient; first, although low, a sufficient concentration of drug has to be present in the tumor, and second, the whole tumor area has to be covered by a permeabilizing electric field. Under these conditions, ECT eliminates the cancer cells while sparing normal cells and histological structures.

### Drugs used in ECT protocols

In late 1980s, Mir et al. [11] described the utility of EP delivery *in vivo* in conjunction with anti-cancer drugs. The requirement for a drug to be used in combination with EPs is first to poorly (or not at all) diffuse through the plasma membrane and second to possess a very high intrinsic cytotoxic potential. Indeed, the ECT principle is to potentiate the drug entrance at the location of the EP delivery while sparing non-electroporated areas. Two anti-cancer molecules have met these prerequisites and are currently used in the clinical practice of ECT, bleomycin and cisplatin [2, 10]. These drugs, once internalized into cells *via* the local delivery of EPs, generate direct DNA lesions, either both single-strand and double-strand DNA breaks (if bleomycin is used) or adducts and intrastrand and interstrand DNA bonds (if cisplatin is used), ultimately leading to cell death. In cells not exposed to EPs, bleomycin enters in very low amounts in a few cell types (e.g., lymphoma cells), possibly through a receptor-mediated endocytotic pathway [12]. After cell electroporation, large amounts of the cytotoxic molecules enter cells by diffusion, regardless of the cell type. Electroporation thus turns bleomycin and cisplatin into very efficient drugs in all tumor types, as verified in preclinical and clinical studies.

Bleomycin and cisplatin administrations, associated with EP delivery, have shown robust anti-cancer activities *in vitro*, *in vivo*, and in humans. *In vitro*, the half maximal inhibitory concentration (IC_50_) of cisplatin was decreased by up to 13-fold when using EPs. Notably, an increased cytotoxicity to the drug was also observed in cisplatin-resistant cell lines [13]. Even more interestingly, the combination of bleomycin with EPs decreased the IC_50_ by up to several hundred folds. The low dose of anti-cancer drug used in ECT protocols, along with the local delivery of the EPs, enables the specific toxicity of the drug toward cancer cells and the absence of systemic side effects. Indeed, both normal and cancer cells are permeabilized in the treated region and thus will get their DNA cleaved by the several hundreds of bleomycin molecules internalized in the electroporated cells in the classical ECT protocols [14]. This number of bleomycin molecules is able to generate enough DNA breaks to drive cell death upon cell division [15] (as is defined, the mitotic cell death) but is not sufficient to drive metabolic cell death. Consequently, normal cells, that are quiescent cells, are spared and systemic side effects are thus rarely observed. In support of this and contrary to many other anti-cancer drugs, no digestive side effects and neither immunosuppression nor myelosuppression have been reported, in particular following systemic bleomycin administration at the specific dose used in ECT protocols. Recently, an alternative to the use of cytotoxic drugs has been proposed through the intratumoral administration of a concentrated calcium solution, associated with EP delivery [16, 17].

### Clinical use of ECT

The first clinical evaluation of ECT with bleomycin was performed in 1991 at Institut Gustave-Roussy (Villejuif, France) in patients affected by head and neck carcinomas [5]. This study confirmed the safety and the feasibility of ECT in humans. Since then, a wide range of tumors has been treated by ECT, mainly using bleomycin [10, 18, 19]. These now include primary tumors (basal cell carcinoma) and metastases of head and neck carcinomas, Kaposi’s sarcoma, breast adenocarcinoma, and melanoma. Clinical trials are nowadays addressing the treatment of deep-seated tumors (primary pancreatic carcinoma and bone, liver, and brain metastases). ECT is particularly indicated to treat bleeding nodules as the treatment immediately stops bleedings and/or hemorrhages. Indeed, ECT demonstrates potent anti-vascular effects [20]. A transient vasoconstriction is observed following EP delivery alone, and moreover, the endothelial cells forming tumor blood vessels are also sensitive to ECT (as any proliferative cell in the treated region). Consequently, these phenomena result in tumor starvation (lack of oxygen and growth factors) and thus contribute to cancer cell death.

In 2006, the multicentric European Standard Operating Procedures of ECT (ESOPE) study [21] established the standard operating procedures for ECT use in the clinic [22]. As reported, a train of eight EPs of 100 μs and of appropriate field amplitude has to be delivered using either invasive (EPs of 1000 V/cm) or non-invasive electrodes (EPs of 1300 V/cm), depending on the depth and on the size of the nodules to treat. This study also reported that a complete tumor regression was observed in 73·7 % of the treated nodules and the overall objective response was 84·8 %, 6 months after one single ECT session. Finally, this study emphasized the safety and the efficiency of the procedure, especially when bleomycin was injected intravenously, although an intratumoral injection can be considered. Contrariwise, due to systemic side effects associated with the intravenous administration of cisplatin, its use in ECT protocols is restricted to intratumoral injections only.

Since the ESOPE study, many other clinical trials have been performed, and overall, the rate of complete tumor regressions following one single ECT treatment is 60 % (85 % of objective response), although this percentage varies among the tumor types [23]. Moreover, it should be noted that after ECT, local relapses are rarely observed [21, 24–26], and if so, the relapse occurs many years later, demonstrating a local long-lasting response to ECT [27].

Side effects of ECT are minimal and include erythema, edema, superficial epidermal erosion, and relative pain and muscle contraction at the time of the EP delivery [23]. As a very low dose of chemotherapeutic is used, these side effects are all due to the EP delivery or to the use of invasive electrodes and are easily manageable by the administration of appropriate anesthesia and myorelaxants.

The only contraindication to ECT regards known allergy to the drug used [22]. Moreover, if the patient has reached a well-known cumulative dose of bleomycin (400,000 IU bleomycin/m^2^ potentially resulting in pulmonary fibrosis), the use of cisplatin has to be considered.

In summary, for the local treatment of superficial tumors of numerous histological origins, ECT is safe (displaying only manageable minor local side effects) [21], rapid (one session lasts about 25 min) [21], and very efficient (85 % of local objective response after one single ECT session) [23]. Development of new electrodes is ongoing in order to apply ECT protocols on tumors located in deep-seated organs, such as colon, liver, bone, and brain, and recent clinical trials reveal that the treatment is also very efficient for these non-superficial tumors [23]. Moreover, from an economic point of view, ECT is a cost-effective treatment that enhances notably the quality of life of the treated patients [28].

Nowadays, ECT is used in routine in about 140 European cancer centers. ECT is used in particular when a palliative treatment of cutaneous and subcutaneous metastases is sought, when tumor burden has to be reduced before surgery or when the surgery would generate non-esthetic scars (e.g., in the head and neck areas) [10, 23]. It is reimbursed in Austria, Denmark, Germany, Italy, Portugal, Slovenia, Spain, Switzerland, and in the UK. Efforts are being made to broaden the range of cancer types treated by ECT, in particular for the treatment of primary tumors. In addition, an increasing number of European oncology centers are adopting this treatment regimen, as reported at the 2010 and 2013 International Users’ Meetings (www.igeamedical.com). By the end of 2015, about 13,000 cancer patients were treated by ECT. Finally, the English National Institute for Health and Care Excellence provided in 2013 interventional procedure guidance, emphasizing the beneficial outcome of ECT as a palliative treatment of skin metastases (www.nice.org.uk).

## Electrogenetherapy

Not only drugs but also nucleic acids can be transferred into target cells *via* the delivery of EPs [4]. The strategy is called gene electrotransfer, and if a therapeutic outcome is desired, it constitutes a subcategory of gene therapy, the electroporation-mediated gene therapy, namely, EGT.

### EGT: gene therapy using electrotransfer

The transfection of target cells by naked DNA only is quite low [29] and variable [30] *in vivo*. Thus, efforts were made to improve the transfection efficiency in order to obtain sufficient protein production. Several strategies are currently available to enhance the transfer of DNA into target cells. One discriminates viral and non-viral gene transfer methods, the latter including chemical and physical methods [7, 8].

Viral vectors are endowed with cell-targeting capacity and are highly effective at transducing target cells. However, the size of the transgene that can be inserted into viral particles is limited; some of these vectors are highly immunogenic (thus preventing multiple administrations); concerns were raised about insertional mutagenesis; and last but not least, GMP-grade viral vectors are expensive to produce.

Contrary to viral vectors, most non-viral vectors are not able to specifically recognize the target cells. Hence, the DNA has to be brought close to the target cell environment, and then, a means to enable the crossing of the plasma membrane is required to bring the DNA into the cells. Chemical vectors are based on cationic lipids or polymers forming complexes with DNA. However, chemical methods are associated with high cell toxicity and low efficiency, in particular *in vivo*. Physical methods (e.g., microinjection, gene gun, sonoporation, and gene electrotransfer) are particularly interesting since large transgenes can be transferred into target cells; the gene expression is transitory and the risk of insertional mutations is dramatically decreased [7]. Gene electrotransfer is one of the most efficient non-viral techniques and has proven its efficiency in many tissues, should they be superficial (e.g., skin) or internal (e.g., liver and muscle). The main limit to the use of gene electrotransfer is related to its application to not easily accessible organs. Finally, gene electrotransfer ensures a local confinement of the DNA [31], preventing the germ line from being transfected and the subsequent transmission of the exogenous genes along generations.

In the context of gene therapy, normal cellular functions can be restored and therapeutic or immunogenic proteins can be endogenously produced. Among the protein-encoding-based gene therapy approaches, the administration of genes encoding growth factors, cytokines, costimulatory proteins, antibodies, or antigens appears quite seducing compared to classical protein therapy [32]. In particular, gene therapy is highly cost-effective as it does not require many administrations and it is associated with a much less variable protein concentration due to a sustained production by the organism. Moreover, the use of naked DNA is very interesting since GMP-grade DNA is much cheaper to produce than GMP-grade proteins. Besides, proteins produced *in situ* are fully functional since the endogenous production ensures the correct folding and post-translation modifications. As for other gene therapy approaches, EGT is a promising method to obtain a therapeutic outcome following the delivery of naked nucleic acids into the cell interior by using gene electrotransfer [33].

### Mechanisms of gene electrotransfer

Gene electrotransfer is a multistep process (Fig. 1c). First of all, DNA has to be injected at the expected site of gene transfer. Then, EPs have to be delivered. Notably, the optimization of the gene electrotransfer procedure enables the use of a reduced quantity of injected DNA to achieve the desired gene expression. Optimally, the gene electrotransfer protocol (field amplitude and duration of the EPs) has to be set up in order for the EPs to achieve two different types of effects [34]. In the first place, EPs have to permeabilize transiently the cell membrane. Then, EPs have to drive electrotrophoretically the DNA toward the electroporated cells. According to one commonly used protocol [35], the combination of short (100 μs) and intense EPs (HV EPs) and long (hundred(s) of milliseconds) and less intense EPs (LV EPs) constitutes a very safe procedure associated with a very good gene expression. The delivery of HV EPs or LV EPs alone is also used in the field of gene electrotransfer and can also be associated with increased gene expression.

Regarding *in vivo* studies, gene electrotransfer has been tested in various animal species (mice, rats, rabbits, and pets) and tissue-specific protocols have been set up for various tissues, including skin, muscle, tumor, liver, cornea, lung, kidney, brain, bladder, and testis [36, 37]. In particular, skin is interesting for vaccination purposes as this organ is densely populated in antigen-presenting cells (APCs), such as dendritic cells (DCs) [38]. However, contrary to muscle, which is considered as a protein factory, transgene expression in skin does not last over months but only over few weeks and at lower levels [35, 36]. Finally, tumor cells may also receive DNA but expression is transient as they divide rapidly and lose the plasmid by dilution over cell divisions. Hence, tumor cell transfection is useful for a short-term production of proteins, such as cytokines exhibiting anti-cancer properties (e.g., interleukin 12).

### EGT: from bench to bedside

Since 1989 and the first approval of a clinical trial involving gene therapy, more than 2000 clinical trials have been launched and more than 60 % of the indications relate to cancer (http://www.abedia.com/wiley). Among the 3700 gene therapy-related clinical trials currently in process, 61 are based on EGT [39]. The two most advanced EGT strategies are related to immunotherapy, namely, DNA vaccination and cytokine-based anti-cancer therapies. We focus on these two specific applications of EGT because of the interest of their association with ECT, as discussed in Sect. 4 of this review.

#### Electroporation-based DNA vaccination

DNA vaccination consists in the administration of a DNA encoding an antigen of interest to protect the body against pathogens or cancer cells exposing this antigen [40]. Eventually, the encoded antigen will be responsible for the generation of a pool of specific B and T cells, from which some will remain as memory cells for long-term protection. Tumor-specific CD8^+^ T cell generation, associated with the secretion of Th1 cytokines (e.g., tumor necrosis factor α (TNFα) and interferon γ (IFNγ)), is desired in the context of anti-cancer therapy [41, 42]. DNA vaccination technology has been developed for a wide range of applications, from laboratory tools to licensed veterinary vaccines.

One of the major factors influencing the DNA vaccination outcome is the level of expression of the antigen administered in its encoding form. Indeed, the poor DNA uptake by the target cells was one of the main reasons that explained the failure in translating to humans the promising results obtained in small rodents with naked DNA administration [40, 43]. Consequently, it is not surprising that EPs are increasingly used for the development of new DNA vaccination strategies [44, 45]. Several studies demonstrated that the immunogenicity of DNA vaccines was greatly increased by electroporation, as compared with DNA vaccine injection alone, in rodent models and in larger animals. A striking example refers to a DNA encoding the prostate-specific antigen (PSA) in the context of prostate cancer [46]. Roos et al. demonstrated that the antigen expression was enhanced by up to 1000-fold when DNA injection was combined with electroporation. This also led to an improved PSA-specific T cell priming. This DNA vaccine is currently being tested in a phase I/II clinical trial (NCT00859729).

Regarding DNA vaccination performed in humans, not less than 51 clinical trials involving electroporation are currently being conducted and about 40 % deal with cancer pathology [39].

#### Electroporation-based cytokine therapy

Interleukin (IL) 12 possesses very interesting properties to fight against cancer [47]. Indeed, it favors CD4^+^ T cell differentiation into Th1 cells; stimulates cytotoxic functions of CD8^+^ T cells, NK cells, and NKT cells, in particular by increasing IFNγ secretion; and possesses anti-angiogenic properties.

Heller and colleagues initiated intratumoral administration in mice of an IL12-encoding plasmid in conjunction with EPs and demonstrated local and systemic anti-tumor effects in a murine hepatocellular carcinoma [48]. Interestingly, the growth of both the treated and distant non-treated tumors was reduced. Moreover, IFNγ production was increased and tumor infiltration by NK cells and T cells was reported. Interestingly, similar observations were made in murine melanoma models [49, 50] with an improved safety as compared with viral IL12 gene therapy [50]. The enhanced tolerability of EGT was explained by a local intratumoral confinement of IL12 when using gene electrotransfer, while IL12 was disseminated into the whole body when using viruses. Indeed, gene electrotransfer resulted in the transduction of no cells other than those located in the volume exposed to the EPs.

Toxicological analysis emphasized the safety of the procedure [51], and other preclinical studies supported the efficiency of IL12 EGT on many other tumor models [52], including in veterinary medicine. In 2008, the first clinical evaluation of an EGT procedure was performed in the context of an IL12 therapy on patients with metastatic melanoma [53]. Apart from the discomfort associated with EP delivery, no systemic side effects were reported. Tumor necrosis and T cell infiltration were observed, and 10 % of patients with non-electroporated distant lesions showed complete regression of all metastases while 42 % displayed a stable disease or partial response. Four phase II clinical studies are currently being conducted in patients with squamous cell carcinomas of the head and neck, metastatic melanoma, Merkel cell cancer, and cutaneous lymphoma (www.clinicaltrials.gov).

## Further links between electroporation-based therapies and immunotherapy

As mentioned in the previous paragraph, EGT strategies are nowadays mainly focused on immune stimulation. Interestingly, there is also compelling evidence that the immune system contributes to ECT efficiency.

### ECT and immunogenic cell death

ECT-mediated tumor regression was dramatically decreased in animals exempt of functional T lymphocytes, in comparison to immunocompetent mice [54–56]. Moreover, the edema observed following EP delivery on tumors (or any other tissue) was more severe in immunocompetent than in immunodeficient mice, consistent with EP-mediated effects that depend on the presence of an intact immune system. The edema, that increases the vascular permeability, probably paves the way for the local infiltrates of DCs [57, 58] and lymphocytes [59] that were found in all ECT-treated tumors starting the next day after the treatment. Gernili et al. showed that ECT treatment of human melanoma led to the maturation of preexisting tumor-resident Langerhans cells, an epidermal subset of DCs, and to their subsequent migration to the tumor-draining lymph node as early as 24 h after the treatment [57]. Similarly, our group detected an intratumoral recruitment of DCs expressing CD80/CD86 maturation markers 48 h after the ECT treatment of immunogenic murine tumors in immunocompetent mice [58]. Sersa et al. demonstrated that an anti-tumor activity of circulating monocytes and splenic T lymphocytes was elicited in mice after ECT treatment of murine SA-1 fibrosarcoma [60]. These studies highlight an immune system activation after the treatment. In a very recent study, we deciphered the mechanisms underlying this immune activation [61]. Indeed, we showed that ECT induces an immunogenic cancer cell death through the liberation of ATP and HMGB1 and the translocation of calreticulin to the cell surface. This immunogenic cell death elicitation is responsible for the generation of tumor-specific T cells [62], potentially able to kill non-ECT-sensitive cancer cells within the primary tumor. Interestingly, cancer stem cells, thought to be responsible for cancer recurrence and metastasis [63], seem sensitive to both extracellular ATP [64] and T cell recognition [65]. Overall, this ECT-driven immune activation might be at least responsible for the absence of local relapses (as frequently reported by the physicians practicing ECT) as well as, potentially, for a limitation of metastatic spreading.

### Adjuvant effect of electric pulses

An increasing number of studies show that delivery of EPs alone to tissues (normal or tumoral) has also some immunological effects [66]. These are briefly summarized below.

EP delivery to tissues, in particular muscles, creates an edema that could facilitate the observed infiltration of macrophages, DCs, and polymorphonuclear leukocytes in the EP-treated volume. It was also found that the expression of classical APC maturation markers, such as the F4/80 antigen and MHC class II molecules, was upregulated once APCs were located in the EP-treated region of the muscles. Consistent with our previous study demonstrating that intracellular ATP is released from electroporated cells [61], it can be hypothesized that EP delivery plays a role of chemoattractant for DCs and their precursors through the *in vivo* ATP release. This release can also favor the differentiation of these precursors and their maturation into DCs with antigen-presenting capacity. It was also postulated that the immune cell recruitment in the electroporated areas originated from the secretion of tumor necrosis factor α, interleukin 1β, and other pro-inflammatory mediators by the electropermeabilized myocytes. Thus, if an immune stimulation is sought through EGT, EPs would be more instrumental than expected if only DNA uptake enhancement is considered.

Consistent with these observations, some authors showed that APC and polymorphonuclear infiltrates in muscles occurred only when DNA injection was coupled with electroporation [67, 68]. This observation was associated with improved immunization in the context of DNA vaccination. Finally, our study also showed that *in vitro* EP delivery on cells leads to calreticulin exposure on the cell surface [61]. As calreticulin acts as a “eat me” signal for DCs [62], it can be speculated that EPs also potentiate the engulfment of the transfected cells by DCs.

APCs, mostly DCs, are of great importance for the outcome of vaccination as they ensure effective T cell priming and maintenance [38]. Therefore, EPs appear to play a pivotal role in anti-cancer DNA vaccination, not only by enhancing the transgene expression but also by recruiting APCs in electroporated tissue areas and by favoring the engulfment of tumor antigens. Eventually, this is responsible for an improved capacity to mount adaptive immune responses against tumors.

### Combination of ECT with immunostimulants

Although ECT is highly efficient on treated nodules, it remains a local treatment having no apparent anti-tumor effects on non-treated distant nodules, even though a CD8^+^ T cell infiltrate has been observed in these latter [69]. Consequently, it is assumed that the anti-tumor immune responses, raised in the context of an ECT-driven immunogenic cell death, are not strong enough to destroy fully established distant tumors. However, preclinical evidence suggests that the association of ECT with immunostimulating agents could be an elegant and efficient way to cure both the ECT-treated nodules and any distant nodule, should it be undetectable metastasis, even in a deep-seated area (Table 1).

**Table 1:** Overview of already tested combination of electrochemotherapy with immunostimulants

Combinations of interest	Species	Tumors	Comparison with ECT alone	References
ECT + recombinant IL2	Mouse	LPB fibrosarcoma	Increased complete regression rate Increased tumor growth delay	[54]
	Human	Metastatic melanoma	Non-comparative study	[73]
ECT + IL2-secreting cells	Mouse	LPB fibrosarcoma 3LL Lewis lung carcinoma	Increased complete regression rate Increased tumor growth delay Systemic effects (i.e., complete regressions) on distant non-treated tumors Anti-metastatic effects Protection against challenge	[69,70]
	Rabbit	VX2 papilloma virus-induced carcinoma	Increased complete regression rate Anti-metastatic effects	[71]
ECT + IL2-encoding plasmid	Mouse	B16 melanoma	Increased survival rate Increased tumor growth delay	[72]
ECT + GM-CSF-encoding plasmid				
ECT + recombinant TNFα	Mouse	SA-1 fibrosarcoma	Increased survival rate Increased tumor growth delay	[74]
	Mouse	SA-1 fibrosarcoma	Increased tumor growth delay Increased tumor necrosis Anti-vascular effects	[75]
ECT + IL12-encoding plasmid	Mouse	SA-1 fibrosarcoma TS/A mammary carcinoma B16 melanoma 4T1 mammary carcinoma SCCVII squamous cell carcinoma	Increased complete regression rate Increased tumor growth delay Increased survival rate Anti-metastatic effects Protection against challenge	[76–78]
	Dog	Various histological origin	Non-comparative study	[80]
	Dog	Various histological origin	Non-comparative study	[79]
ECT + CpG oligonucleotides	Mouse	B16 melanoma LPB fibrosarcoma	Increased complete regression rate Systemic effects (i.e., complete regressions) on distant non-treated tumors Increased tumor growth delay	[58]
ECT + ipilimumab	Human	Metastatic melanoma	Non-comparative study	[85]
	Human	Metastatic melanoma	Non-comparative study	[84]

Pioneer works combining ECT with IL2-based immunotherapy led to promising results. IL2 is a T cell proliferation factor and a cytokine with tumor growth inhibition properties. Recombinant IL2 administration in combination with ECT treatment of murine LPB sarcoma tumors led to an increased rate of tumor cures, as compared with ECT alone [54]. More strikingly, in a two-tumor model, ECT was combined with an intratumoral administration of histocompatible IL2-secreting cells. This resulted in an increased efficiency of the treatment in the ECT-treated LPB tumors but also generated a systemic response as anti-tumor effects were observed in the contralateral non-ECT-treated tumors [69]. Actually, when the combined treatment was used, contralateral non-ECT-treated tumors were highly infiltrated by CD4^+^ and CD8^+^ T lymphocytes, probably responsible for the observed 50 % tumor rejection rate of these untreated contralateral tumors [69]. Similar protocols resulted in anti-metastatic effects following the treatment of subcutaneous murine 3LL Lewis lung carcinoma [70] or VX2 papilloma virus-induced carcinoma transplanted in rabbit’s liver [71]. Besides, a long-term anti-tumor protection against recurrence and challenge was conferred in mice by granulocyte-macrophage colony-stimulating factor or IL2 gene electrotransfer, in association with ECT treatment of B16 tumors [72]. In humans, the combination of ECT and IL2 administration was tested in melanoma patients and resulted in an increased number of antigens recognized by specific tumor-infiltrating T cells [73]. Although not verified in the study, one can assume that this T cell response resulted in cancer cell death within the tumors. Apart from the IL2-based immunotherapies, TLR9 ligands (e.g., CpG oligodeoxynucleotides), known to induce Th1 immune responses, were also tested and their injection into ECT-treated tumors dramatically increased the treatment efficiency in immunocompetent mice [58]. The ECT/CpG combination also revealed systemic anti-tumor effects since tumor growth delays and tumor rejections were observed in contralateral non-treated tumors. These effects were even more drastic in highly immunogenic LPB tumors than in less immunogenic B16 melanoma tumors. No such systemic effects were observed when ECT alone was applied. The systemic effects relied, at least partially, on T cell-mediated immune responses since no effects were observed in nude mice. Furthermore, Sersa et al. showed that TNFα administration associated with ECT treatment of murine SA-1 fibrosarcoma also presents interesting benefits, in particular by increasing tumor necrosis and inducing anti-vascular effects [74, 75]. The same group showed that the combination of ECT with intramuscular IL12 gene electrotransfer leads to improved cure rates of SA-1 tumors and TS/A mammary carcinoma in mice [76]. Similarly, Kishida et al. [77] and Torrero et al. [78] concomitantly treated murine B16 melanoma, 4T1 mammary carcinoma, or SCCVII squamous cell carcinoma with ECT and intratumoral IL12 gene electrotransfer. Both studies reported an increased efficacy of the treatment, an inhibition of metastasis development, and a prolonged survival, in comparison with the single-treatment modalities. These findings were successfully translated in dogs with spontaneous neoplasms from various origins [79, 80]. In conclusion, the combination of ECT with immune stimulation is a very promising avenue for complete and long-term cancer eradication.

## Perspectives: immunotherapy, an effective way to render systemic the efficacy of the ECT

**Fig. 2:**
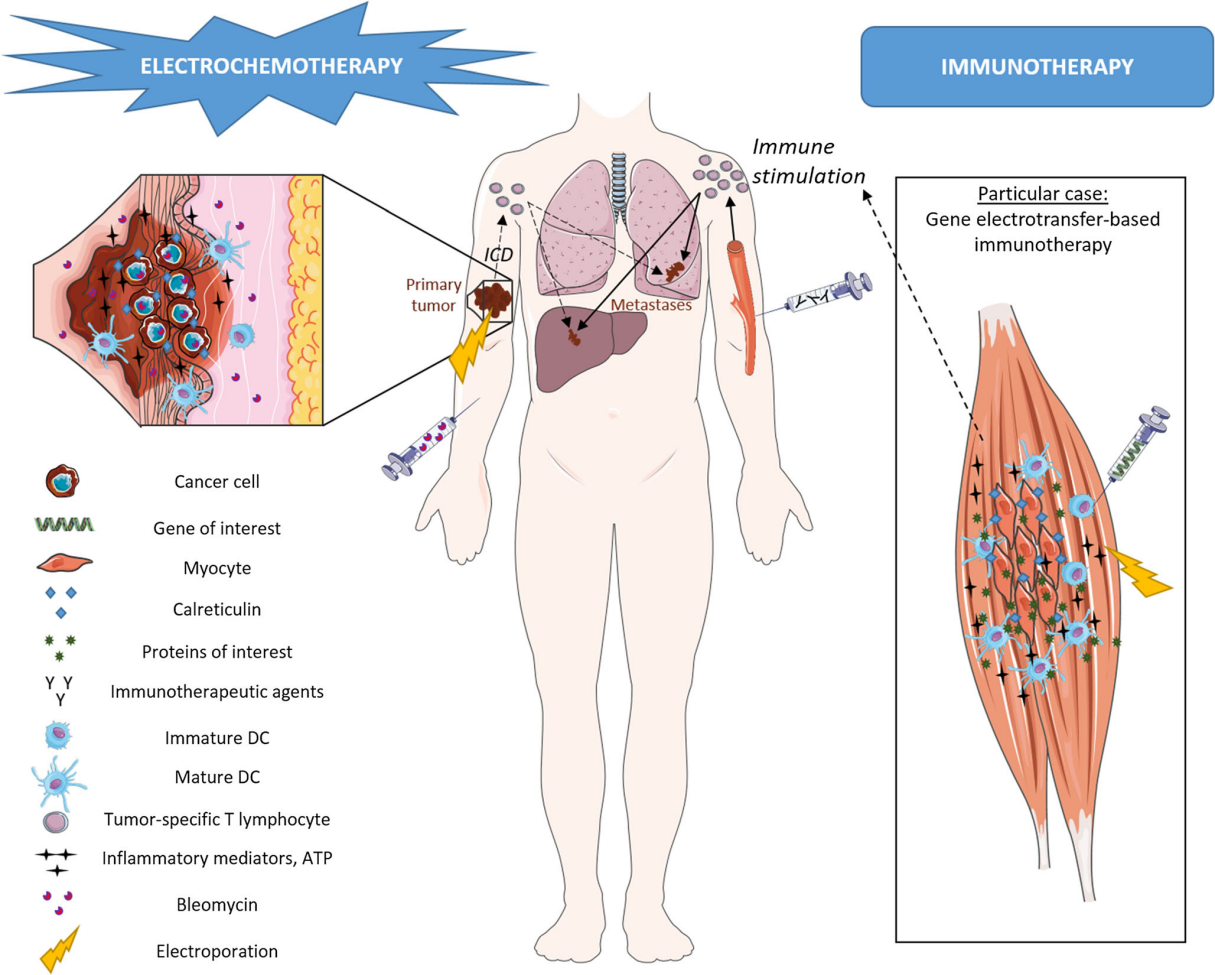
Combination of anti-tumor electrochemotherapy with immunotherapy for long-term and systemic anti-tumor responses. *left* Anti-tumor ECT consists in the injection of non- or low-permeant anti-cancer drugs, such as bleomycin and cisplatin, followed by electroporation to enhance cell permeability. Because of the direct cytotoxicity of the drug toward dividing cells, most cancer cells are driven into death. The remaining viable cancer cells within the treated tumor can be destroyed by tumor-specific T cells, primed in the context of ECT-mediated immunogenic cell death (ICD). Theoretically, these tumor-specific T cells can also target metastatic nodules, although there is a lack of direct evidence in the absence of a complementary immune stimulation. *right* Immunotherapy agents (e.g., cytokines, therapeutic antibodies, immune checkpoint blockers, and genes) mount immune responses that could potentially be synergistic with the one triggered by ECT. More specifically, immunostimulating EGT triggers specific (DNA vaccination) or unspecific (cytokine-based EGT) immune responses against cancer cells leading to their eradication, no matter where they are located in the body. In all, the combination of ECT with immunotherapy, including those based on EGT, is an elegant strategy to treat both the primary tumors and to kill any other cancer cells in the body

ECT is a very efficient local anti-cancer treatment used for superficial lesions and being evaluated for deep-seated ones [2, 23]. It is a very effective tumor-debulking approach displaying immunostimulating properties through immunogenic cell death elicitation [61]. It is considered as a very safe treatment and is increasingly used in Europe for the palliative treatment of cutaneous and subcutaneous cancerous nodules. Although a long-lasting response is usually observed locally, no distant anti-tumor effects in untreated nodules are observed following ECT treatment alone. Interestingly, preclinical results sustain the use of immunotherapy in conjunction with ECT to obtain complete and long-term cancer eradication. Noteworthy, some recent immunotherapy approaches have shown promising results for cancer treatment and constitute seducing approaches to restore or strengthen anti-tumor immunity [81]. For example, ipilimumab is a monoclonal antibody administered to patients with metastatic melanoma [82], a tumor extensively studied in the field of ECT. Ipilimumab is directed against cytotoxic T lymphocyte-associated protein 4 (CTLA4) on T cells which turns down the T cell effector functions. Similarly, anti-programmed cell death protein 1 (PD1) antibodies are also of great interest as they prevent the inhibitory effect on T cell functions of the interaction between PD1 (on T cells) and PD1 ligand (on tumor cells) [83]. Hence, a combination of ECT with anti-CTLA4 or anti-PD1 antibodies could be an elegant way to destroy the initial nodule while raising efficient anti-tumor responses to ultimately eliminate remaining and circulating cancer cells. In this spirit, two recent studies seeking to evaluate the potency of the combination of ipilimumab and ECT showed very encouraging results [84, 85]. In the case report published by Brizio et al. [84], local ECT treatment of cutaneous lesions of melanoma was followed by ipilimumab administration, resulting in the complete regression of all the cutaneous and visceral metastases for at least 1 year. Interestingly, vitiligo-like lesions developed exclusively around the sites of previous ECT, suggesting that a prior ECT-driven immune activation was enhanced by ipilimumab. The other study reported that the volume of distant non-ECT-treated tumors decreased or was stabilized in nine patients out of 15, possibly through ipilimumab-induced regulatory T cell depletion [85]. Of course, as presented in the previous paragraph, ECT could also be associated with other immunostimulating agents, such as cytokines with anti-tumor properties. Alternatively, immune stimulation through EGT has also recently raised great hope for the treatment of cancer [53]. EGT appears safe, and besides their ability to enhance the DNA uptake, EPs may potentiate the beneficial outcome of immunostimulating EGT, notably of DNA vaccination, through immunological adjuvant effects [66]. Further studies are urgently needed to confirm the valuable therapeutic potential of all these combinations. It is conceivable that a combination of tumor-debulking ECT with immunostimulating EGT could get the patients rid of not only the primary tumors and accessible metastases (like by using surgery) but also all of the non-accessible metastases and micrometastases, thus hampering cancer chronicity (Fig. 2). *ECT* electrochemotherapy, *IL* interleukin, *GM-CSF* granulocyte-macrophage colony-stimulating factor, *TNF* tumor necrosis factor.
